# Estimating Parameters Related to the Lifespan of Passively Transferred and Vaccine-Induced Porcine Reproductive and Respiratory Syndrome Virus Type I Antibodies by Modeling Field Data

**DOI:** 10.3389/fvets.2018.00009

**Published:** 2018-01-29

**Authors:** Mathieu Andraud, Christelle Fablet, Patricia Renson, Florent Eono, Sophie Mahé, Olivier Bourry, Nicolas Rose

**Affiliations:** ^1^Unité épidémiologie et bien-être du porc, Anses Laboratoire de Ploufragan-Plouzané, Ploufragan, France; ^2^Université Bretagne-Loire, Rennes, France; ^3^Unité Virologie Immunologie Porcines, Anses Laboratoire de Ploufragan-Plouzané, Ploufragan, France; ^4^Union des Groupements de Producteurs de Viande de Bretagne (UGPVB), Rennes, France

**Keywords:** vaccine-induced immunity, maternally derived antibodies, parameter estimation, porcine reproductive and respiratory syndrome, antibody kinetics

## Abstract

The outputs of epidemiological models are strongly related to the structure of the model and input parameters. The latter are defined by fitting theoretical concepts to actual data derived from field or experimental studies. However, some parameters may remain difficult to estimate and are subject to uncertainty or sensitivity analyses to determine their variation range and their global impact on model outcomes. As such, the evaluation of immunity duration is often a puzzling issue requiring long-term follow-up data that are, most of time, not available. The present analysis aims at characterizing the kinetics of antibodies against Porcine Reproductive and Respiratory Syndrome virus (PRRSv) from longitudinal data sets. The first data set consisted in the serological follow-up of 22 vaccinated gilts during 21 weeks post-vaccination (PV). The second one gathered the maternally derived antibodies (MDAs) kinetics in piglets from three different farms up to 14 weeks of age. The peak of the PV serological response against PRRSv was reached 6.9 weeks PV on average with an average duration of antibodies persistence of 26.5 weeks. In the monitored cohort of piglets, the duration of passive immunity was found relatively short, with an average duration of 4.8 weeks. The level of PRRSv-MDAs was found correlated with the dams’ antibody titer at birth, and the antibody persistence was strongly related to the initial MDAs titers in piglets. These results evidenced the importance of PRRSv vaccination schedule in sows, to optimize the delivery of antibodies to suckling piglets. These estimates of the duration of active and passive immunity could be further used as input parameters of epidemiological models to analyze their impact on the persistence of PRRSv within farms.

## Introduction

Mathematical models represent powerful tools to understand the spread of infectious diseases in humans and animals, providing informative insights to stakeholders for knowledge-based decision making. In addition to an adequate model structure representing the epidemiological processes accurately, the outcomes are strongly dependent on the reliability of the parameter sets. The parameters are often based on the fit of theoretical model to field or experimental epidemic data (1). However, the estimation for some parameters may require specific, and often unavailable, data. In this case, uncertainty or sensitivity analyses are commonly performed to assess the variation range of these parameters and their relative impact on the course of infection (2–6). One example relies on the evaluation of the duration of vaccine-induced or post-infection immunity, requiring long-term longitudinal follow-up studies which are difficult and costly to set-up.

Porcine Reproductive and Respiratory Syndrome virus (PRRSv) is a small RNA virus responsible for large economic losses in pig industry throughout the world (7–9). Controlling PRRSv infection in endemically infected farms is therefore of primary importance for swine producers. Vaccination appears as a potential solution to address this issue. Different vaccination schemes, using modified live vaccine (MLV), targeting either breeding animals or growing pigs, are therefore currently used in the field. Sow vaccination aims at reducing the clinical consequences of the infection and helps stabilize the infection in the breeding herd, limiting the viral transmission between individuals and providing sufficient amount of maternally derived antibodies (MDAs) to their offspring. For the latter purpose, batch-targeted vaccination, where all sows within a batch are vaccinated about 4 weeks prior to farrowing, should theoretically induce high and homogeneous MDA levels in newborns. In contrast, mass vaccination, where all breeding sows are simultaneously vaccinated every 4 months, reduces the infectious pressure to limit the spread of the pathogen among sows and potential vertical transmission to embryos or newborns. Although vaccination has a strong impact on reproduction troubles related to PRRSv infection, the epidemiological benefits in terms of reduction of the transmission of the virus are less convincing in field conditions (10, 11). Two recent experimental studies estimated reproduction numbers lower than one in vaccinated animals (12, 13). Transposing these results to the field would theoretically lead to PRRSv eradication following vaccination in chronically infected herds, which is obviously not the case as the disease remains enzootic in many densely pig populated areas throughout the world. The role of MDA on PRRSv epidemiology is also puzzling. Passive immunity protection is undoubtedly essential for piglets in the early life, but this protection is known to be partial, limiting but not avoiding transmission and short-termed. Therefore, MDA could also have adverse effects on PRRSv epidemiology, favoring the persistence of the virus within the herd as was recently evidenced for swine influenza (5). Furthermore, passive immunity may also interfere with the vaccination of growing pigs, impairing the onset of effective immune response with potential side-effects in terms of virus transmission (14).

The immune response against PRRSv is therefore a major factor modulating the course of infection (15), and increasing knowledge on the role of immunity on the epidemiological processes appears essential. The durations of both active and passive immunities are pivotal and not well known parameters impacting the frequency of reinfections and the age at first infection, respectively. Feeding epidemiological models with robust parameter estimates will further help to analyze the global spread and persistence of PRRSv within farms and to schedule adequate vaccination programs within herd. The present analysis aims at characterizing the kinetics of antibodies against Porcine Reproductive and Respiratory Syndrome virus from two available longitudinal data sets on post-vaccination (PV) PRRSv antibody response in gilts and MDA kinetics in piglets. The serological response, evaluated through an ELISA detecting anti-nucleocapsid protein antibodies, even not representative of immune protection, was chosen as an easy-to-access indicator fully available for practitioners to provide them with useful information for field applications.

## Materials and Methods

### Data

The study was performed in accordance with current legislation on ethical and welfare recommendations. ANSES-Ploufragan is certified for animal experimentation and is registered under certification number C-22-745-1 delivered by the official French veterinary services.

#### Vaccine-Induced Immune Response in Breeding Animals

Serological data from a longitudinal follow-up of two successive batches of 11 gilts (further denoted G1 and G2) were considered. The animals, derived from a PRRSv-free herd, were vaccinated (Porcilis^®^ PRRS, MSD) twice with a 3-week interval schedule (intramuscular route). Individual blood samples were taken on the day of first vaccination (*T*_0_), to ensure the absence of PRRSv-specific antibodies. Gilts in group G1 were further sampled on weeks 3, 5, 7, 10, 17, and 21 post first vaccination (PFV). In group G2, blood samples were collected on weeks 1, 3, 6, 8, 10, 17, and 20 PFV. Samples were all held back and tested at one time using a single assay lot. Evolution of the serological titers after vaccination was determined using the PRRS X3 Ab ELISA kit (IDEXX, Liebefeld-Bern, Switzerland) with a seropositive cut-off S/P ratio of 0.4. Even if this test is not a true quantitative method, there is a good correlation between S/P value and antibody titer, except for very low or high levels (Bourry, unpublished data).

#### MDAs and Infection Kinetics in Piglets

Data were derived from a longitudinal study in pig populations (16). Briefly, 40 pigs per batch from 3 successive batches were individually monitored from birth to slaughter age in 3 different herds known to be chronically infected by PRRSv (n = 360 pigs in total). One week post-farrowing, 4 piglets born to 10 randomly selected sows were chosen at random in each batch and ear tagged. At the same time, blood samples were collected from the sows. Blood samples were also taken from these piglets at 1, 6, 10, 14, 18, and 22 weeks of age. Selected herds were known to vaccinate breeding animals against PRRSv (Porcilis^®^ PRRS, MSD) but growing pigs were not vaccinated. Samples were all held back and tested at one time using a single assay lot. Sera were analyzed using the Idexx (HerdChek*) PRRS X3 Ab ELISA kit previously mentioned to obtain the evolution of individual serological profiles with age.

### Mathematical Models and Parameter Estimation

#### Vaccine-Induced Serological Response in Gilts

Visual inspection of individual serological profiles evidenced a rapid onset of the antibody response with a peak around 3 weeks PV, followed by an exponential decay. Therefore, the PV antibody kinetics was fitted to Wood’s function, according to which the S/P ratio *A_ij_* of individual *i* at observation time *t_j_* is given by:

$$A_{\mathrm{ij}}=a_{i}t^{b_{i}}\exp(-c_{i}t_{j})+\varepsilon_{\mathrm{ij}},\text{ where }\varepsilon_{\mathrm{ij}}\sim N(0,\sigma )$$

where *a_i_, b_i_*, and *c_i_* are the individual parameters governing the Wood’s function, ε*_ij_* is the residual error for the *j*^th^ observation in subject *i*, and σ is the variance of the residual unidentified variability (17). Uniform prior distributions were used $\left(a_{i}\sim U(0,3),b_{i}\sim U(0,5),c_{i}\sim U(0,1)\right)$ for Wood’s function parameters, and parameter estimation was performed using the Metropolis Hasting algorithm implemented in R (18). Three independent chains were run with randomly chosen initial values drawn from prior distribution. Each chain consisted of 55,000 iterations including a burn-in period of 5,000 iterations with a thinning interval of 10 iterations. Convergence was assessed through visual inspection and convergence diagnostic tests (Heidelberger, Geweke, Gelman-rubin tests). The duration of vaccine-induced immunity was estimated from predictions of individual antibody kinetics based on individual parameter estimates (19). Waning of PRRSv antibodies was considered when the predicted individual serological titer fell below the seropositive cut-off S/P ratio defined by the test manufacturer (0.4).

#### MDAs Kinetics

To analyze the early life antibody kinetics in piglets, we focused on animal serological profiles from birth to 14 weeks of age. During this time period, most animals that were initially MDAs positive evidenced an exponential decay of their S/P ratio. However, a few of these individuals showed a sudden increase of their antibody titers, presumably linked to an infection albeit being passively immune. For these individuals, the profiles were limited to the decreasing phase of antibody titers. The kinetics of MDAs waning was modeled as described in (16). Briefly, a nonlinear mixed effect model assuming an exponential decay of antibody titers was used (17). Two parameters were estimated: the initial serological titer *A*_0_ and the decay rate *r*. As the initial antibody titer was deemed being closely linked with the dam serological titer, the latter was considered as a covariate acting on *A*_0_. Thus, the model describing the serological titer of individual *i* at observation time *t_j_* (with a constant residual error model) is given by:

$$A_{\mathrm{ij}}^{\;}=A_{0}^{\left(i\right)}\exp(-r_{i}\;t_{j})+a\varepsilon_{\mathrm{ij}},$$

where $A_{0}^{\left(i\right)}$ and *r_i_* are the individual parameters and ε*_i_* is a vector of standardized random variables. As described in Ref. (20), individual parameters were assumed to be log-normally distributed. The parameters for individual *i* are given by:

$$\begin{array}{l} \log(A_{0}^{\left(i\right)})=\text{log}(A_{0}^{\left(\text{pop}\right)})+{\text{β}}_{\text{Dam}}\log(A_{\text{Dam}})+{\text{η}}_{A}^{\left(i\right)}\text{ and }\\ \;\log(r_{i})=\text{log}(r_{\text{pop}})+{\text{η}}_{r}^{\left(i\right)} \end{array}$$

where $r_{\text{pop}}$ denotes the median decay rate at the population level. $A_{0}^{\left(\text{pop}\right)}$ represents the mean initial serological titer in the population. ${\text{η}}_{A}^{\left(i\right)}$ and ${\text{η}}_{r}^{\left(i\right)}$ are vectors of random effects assumed as independent centered Gaussian vectors with variance ${\text{ω}}_{A}^{2}$ and ${\text{ω}}_{r}^{2}$, representing inter-individual variability. Individual projections were performed using the individual parameter estimates to determine the waning age of MDAs (19).

## Results

### Vaccine-Induced Antibody Response in Breeding Animals

**Figure 1:**
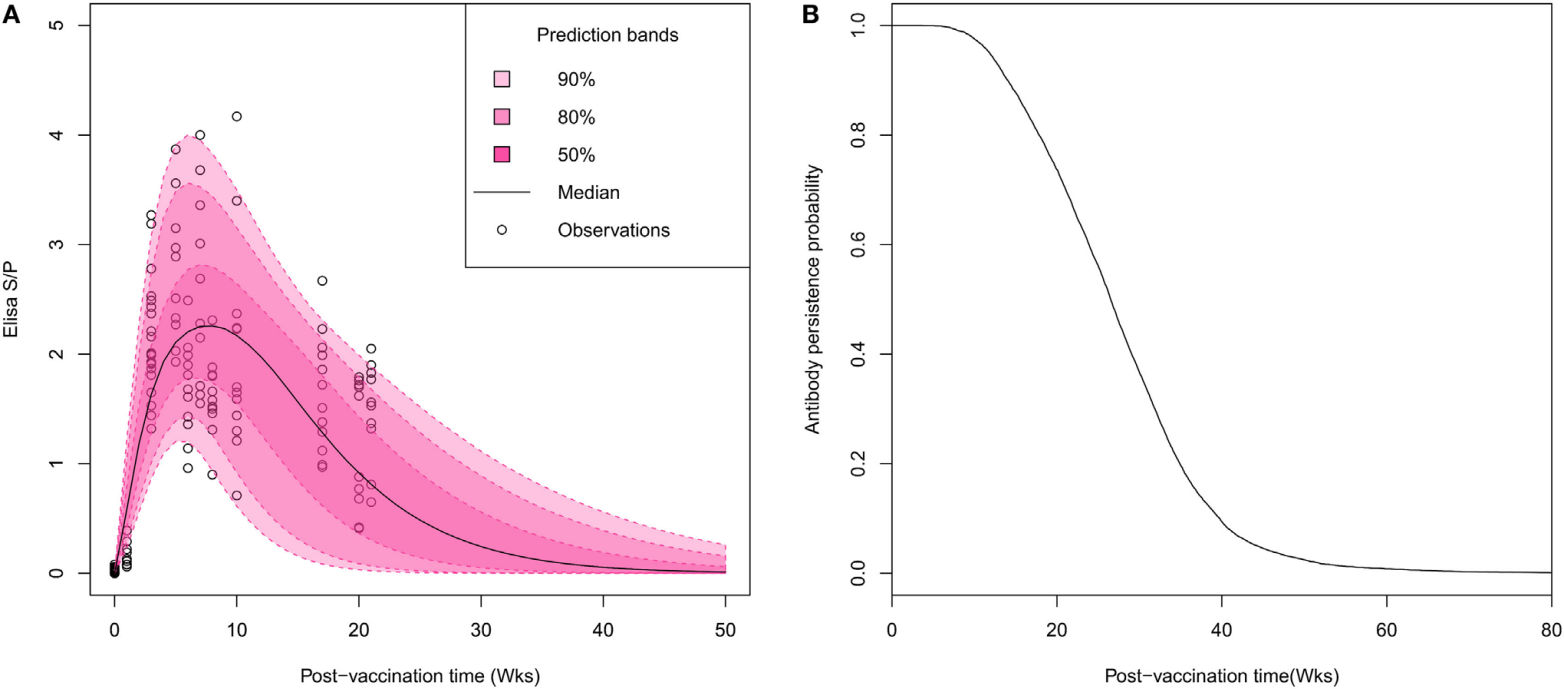
Characterization of the duration of vaccine-induced immunity in breeding animals. **(A)** Visual predictive distribution of antibody kinetics. Pink areas and the black line correspond to percentiles and median of predicted values based on estimated individual parameters. **(B)** Gamma distribution of survival times of vaccine-induced antibody persistence. The average duration of antibody persistence was estimated to 26.5 weeks with inter-individual variability ranging between 10 and 49 weeks.

Visual inspection of individual serological profiles after vaccination evidenced a rapid onset of the immune response, all gilts having antibody titers beyond the test threshold (S/P ratio > 0.4) on week 3 PV (Figure 1A). Thus, the estimation of Woods’ function parameters permitted to figure out the large inter-individual variations in both intensity and durations of vaccine-induced antibody response. The peak of antibody response was reached on average 6.9 weeks PV (95% credibility interval: 3.3–13.2). Half of vaccinated animals reached their highest antibody titer between the fifth and the ninth week PV with S/P ratios ranging from 1.3 to 4.2 (mean 2.5). The individual antibody kinetics were predicted from the individual parameter estimates to determine the time at which each individual profile crossed the test threshold value. The median time of vaccine-induced antibody waning was evaluated to 26.5 weeks with large variations between individuals (95% CI: [10; 49]; Figures 1B).

### MDAs Kinetics in Piglets

**Figure 2:**
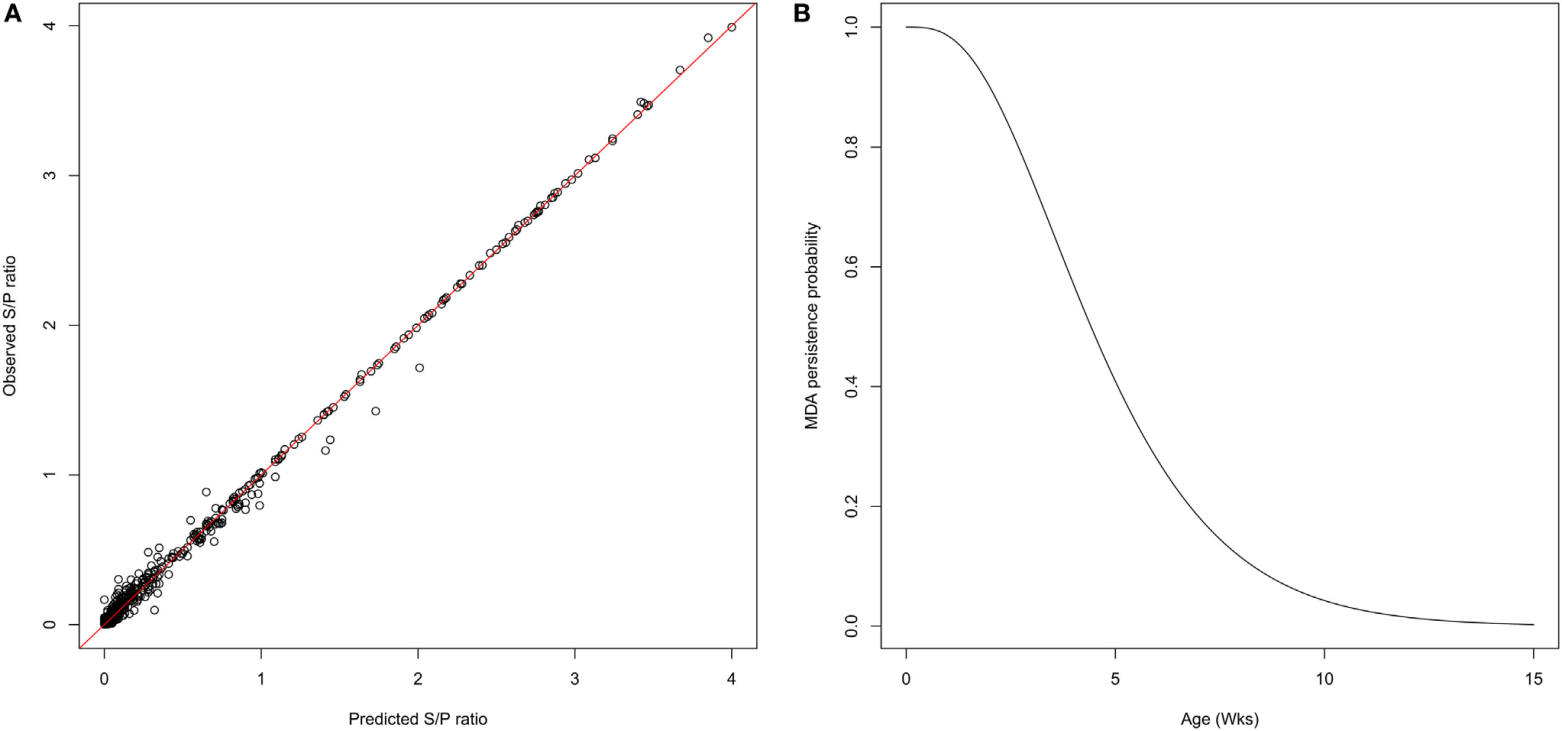
Characterization of MDA persistence. **(A)** Predicted versus observed S/P ratio showing a goodness of fit obtained with the exponential model decay used to represent the MDA kinetics (*R*^2^ = 0.98). **(B)** Gamma distribution of survival times of MDA persistence. The average duration of antibody persistence was estimated to 4.8 weeks, 90% of individuals being seronegative at 10 weeks of age.

One week after farrowing, 83% of sows were found positive for PRRSv-specific antibodies, potentially able to deliver MDAs to their offspring with an average S/P ratio ranging from 0.5 to 4.2. Thirty piglets showed a serological response with a sudden increase of antibody levels, likely due to infection, while still having MDAs above the test threshold. Furthermore, for the sake of parameter identifiability, only individual profiles with at least three successive decreasing datapoints were considered for the estimation of the parameters governing the antibody kinetics. A total of 227 individual profiles were finally included in the analysis. The average initial S/P ratio in piglets was estimated to 1.9 with relatively large inter-individual variability $\left({\text{ω}}_{A}=0.34\right)$. At the individual scale, the initial MDA S/P ratio was found significantly correlated with the S/P ratio of their dam (*β*_Dam_ = 0.27, *p* < 0.001; Table 1). The antibody decay rate was estimated to 0.33 per week, with a weak variability among the individuals $\left({\text{ω}}_{r}=0.04\right)$. Empirical Bayes estimates diagnostics were used to assess the model adequacy. Visual inspection of the individual predictions revealed a good adequacy of the model to data (Figure 2A, *R*^2^ = 0.98, ε-shrinkage: 21%). η-shrinkages were also found below 30% (2 and 17% for parameters *A*_0_ and *r*, respectively), showing that the level of information provided by the data was sufficient for individual parameter estimation.

**Table 1:** Parameter estimates of PRRSv MDAs kinetics.

	Parameter estimate	SE	*p*-Value
*A*_0_	1.9	0.34	
*r*	0.33	0.07	
*β*_Dam_	0.27	0.03	<10^−3^

*A_0_: initial antibodies titer (S/P value of the ELISA test)*.

*r: antibody decay rate (day^–1^)*.

*β_Dam_: sow’s serological titer covariate coefficient on piglets’ initial antibody titer*.

The individual predictions of the antibody kinetics were further used to evaluate the duration of passively transferred antibodies waning (assuming a threshold value of 0.4 S/P ratio). The durations of MDA persistence was fitted to a gamma distribution (shape = 3.60 [3.08; 4.17]; scale = 1.35 [1.14; 1.59]), leading to a mean duration of 4.8 weeks, more than 90% of individuals being MDA negative at 10 weeks of age (Figure 2, right panel).

## Discussion

Robust parameterization of epidemiological models is essential for reliability of output interpretation. Estimation of parameters regarding pathogens transmission and population immunity allows feeding the model using external data based on experimental and field studies. The present study proposed the analysis of antibody kinetics from longitudinal data sets to gain insights into the characteristics of the immune response. The methodology was applied to two serological data sets describing the antibody kinetics regarding PRRSv in vaccinated gilts and newborn piglets, respectively. Porcine reproductive and respiratory syndrome is a major economic issue for swine industry, for which the immune component is known to play a major role on the disease spread. The serological data were obtained using a commercial ELISA test [(HerdChek*) PRRS X3 Ab kit], which is most commonly used in the field. Although not directly related to a functional protective immunity, the induction of antibodies detected by the ELISA after vaccination was shown to be associated with the appearance of the cell-mediated immune (CMI) response, which is a key player of the protective immunity against PRRSv. Indeed we recently showed in vaccinated piglets that the seropositive animals identified by ELISA developed a CMI response, whereas in the piglets that did not seroconvert, no CMI was detected (14). The use of modified live vaccines toward PRRSv ensures an immune response close to the one developed in case of wild infection, making the results transposable to the natural infectious process within endemically infected farms.

The first data set described the antibody kinetics after PRRSv vaccination in breeding animals. Visual inspection of individual serological profiles after vaccination evidenced a rapid onset of immune response, supporting previous observations (21), followed by exponential decay of antibody titers with relatively large inter-individual variations in both intensity and durations of vaccine-induced immunity. The antibody kinetics was described using an analytical function, e.g., the Wood’s function, which was also used to represent the viral kinetics of PRRSv (22). This function, originally used to describe the lactating curves in cows, presents similar characteristics as the observed antibody kinetics but tends to 0 as time gets larger. Such an assumption, well suited for modeling viremia profiles, may appear rather strong for representing the serological response, artificially implying immunity waning for all individuals. However, the animals involved in our study were PRRSv free before vaccination and were not exposed to external infection pressure after vaccination. In this specific context, persistence of humoral immunological response due to booster consecutive infections is unlikely, and immunity waning is presumed as the most probable outcome. These antibodies were found to persist up to 1 year, with an average persistence of approximately 6 months (26.5 weeks). The peak of PV antibody production was found to occur on average 6 weeks after vaccination, with a rather large credibility interval [3.3; 13.2]. Half of the vaccinated gilts reached their highest antibody S/P ratio between the fifth and the ninth weeks PV. This time window before parturition represents vaccination schedule offering the optimal MDA level to newborn piglets at birth. Indeed, from the data set based on growing pigs antibody kinetics, the initial antibody titers of the newborns were found positively correlated to those of their respective dams around parturition time. In this study, 83% of piglets had MDA, in line with the observed seroprevalence in sows at 1 week post-parturition. Two batches from the same herd showed seroprevalences below 70% at 1 week of age despite high seropevalences in sows in these specific batches. These low seroprevalences might result from several external factors influencing colostrum intake by piglets that had not been accounted for in our study, such as litter sizes, birth weights, and order or piglets vitality traits (23–25). Among MDA positive piglets, 10% showed an increase of their serological titer while still having passive immunity. Although protecting the animals from clinical consequences, passively acquired immunity only confers a partial and temporary protection toward PRRSv. Furthermore, the interference between MDAs and vaccination was evidenced for several pathogens, with potential detrimental consequences on further wild infection occurrence (26–30). PRRSv does not make an exception, as a recent study evidenced an impaired immune response to vaccination in animals harboring neutralizing antibodies (14). The duration of passive immunity is therefore an essential factor to establish an optimal vaccination schedule. The kinetics of neutralizing antibodies sharing similar characteristics as Elisa-detected antibodies (14), the present results could be extended to the persistence of vaccine-induced and maternally derived neutralizing antibodies.

The antibody decay rate was estimated to 0.33 per week, corresponding to a half-life antibody concentration of 2 weeks, in line with earlier estimates (31, 32). Based on individual predictions of antibody kinetics, passive immunity was estimated to last on average 4.8 weeks. This duration appears relatively short in contrast with the durations of passive immunity for other infectious diseases, as swine influenza or hepatitis E, for which the durations were estimated to 7 and 10 weeks, respectively (16, 33).

The present work aimed at characterizing the antibody kinetics in vaccinated sows and newborn piglets with application to PRRSv. Although presenting some caveats, such as the low number of involved gilts, data from a unique herd or the use of one vaccine, our analysis allowed for the estimation of targeted parameters and could be considered as a supervised learning example for further studies. The methodological approach, here applied to describe analytically the antibody kinetics, could be adapted, through the representation of mechanistic processes, to alternative immune parameters whenever quantitative data would be available. The estimation of the duration and intensity of vaccine-induced immunity in breeding animals could be further used to feed epidemiological models. Knowledge-based parameterization increases the robustness of model outcomes, allowing for better representation of actual epidemiological processes. This is particularly the case for the interplay between immunity and transmission of PRRSv. The occurrence of reinfections after immunity waning and infections in the presence of MDA are deemed to favor the persistence of the virus within farms, but their impact on the viral spread still need to be objectivized.

## Ethics Statement

The study was exempt from ethic statements. No data were specifically collected for the study focusing on the analysis of historical data.

## Author Contributions

MA developed the mathematical model, performed data analysis, and drafted the manuscript. NR conceived and supervised the study. CF supervised the field study and analyzed the data. PR, SM, and FE performed laboratory analyses. OB supervised laboratory analyses. All co-authors revised the manuscript and approved the final submitted version.

## Conflict of Interest Statement

The authors declare that the research was conducted in the absence of any commercial or financial relationships that could be construed as a potential conflict of interest.
